# Clinical Efficacy and Safety of a Highly Purified Polynucleotide for Dry and Chapped Lips: A Prospective, Multicenter Study

**DOI:** 10.1111/jocd.70224

**Published:** 2025-05-22

**Authors:** Nark Kyung Rho, Hyun Jun Park, Hei Sung Kim

**Affiliations:** ^1^ Leaders Aesthetic Laser and Cosmetic Surgery Center Seoul Korea; ^2^ Maylin Clinic Cheongdam Seoul Korea; ^3^ Department of Dermatology, Incheon St. Mary's Hospital College of Medicine, The Catholic University of Korea Seoul Korea

**Keywords:** dry and chapped lips, polynucleotide, prospective, roughness, vermilion, wrinkle

## Abstract

**Background:**

Dry and chapped lips are a common nuisance. Besides the discomfort, many report feeling embarrassed over the unsightly appearance of their chapped lips. The aim of this study was to determine if polynucleotide (PN) injection helps relieve lip dryness and assess safety in practicing PN in this specific area.

**Methods:**

In this prospective study, 30 Korean subjects with dry and chapped lips enrolled to receive PN injection on the vermilion zone (a total of 3 injections, each 3 weeks apart). Vermilion wrinkle and roughness were scored based on the photos taken pretreatment (week 0); before the 2nd and 3rd injection (week 3, week 6); and 3 weeks after the 3rd injection (week 9) using the respective severity rating scales. Safety data were obtained throughout the study period.

**Results:**

A total of 27 individuals completed the study. PN significantly improved both the vermilion wrinkles (assessed by the Wrinkle Severity Rating Scale (WSRS)) and roughness (assessed by the Lip Roughness Grading Scale (LRGS)) at all time points compared to baseline (*p* < 0.05). The WSRS response rate (Ratio of subjects whose WRSR decreased by ≥ 1 point compared to baseline) at week 9 was 100%. Lip swelling was experienced by all subjects when examined 30 min after PN injection, followed by pain (90%) and redness (77%). The injection site adverse reactions were mostly mild and transient.

**Conclusion:**

Our results suggest that PN may be a promising option to soothe dry and chapped lips. However, larger studies with long‐term follow‐up are necessary to confirm the preliminary findings of this study.

## 1 Introduction

Lips are unique oral structures and their appearance has a significant impact on facial aesthetics. Both the upper and lower lip comprise 3 distinct zones: the cutaneous surface (labial skin), the vermilion, and the mucosal membrane (labial oral mucosa) [1]. The vermilion zone is distinctly different from the surrounding skin with its characteristic rosy color [2]. As the vermilion is often referred to as “the lip”, the term “lip” will be used interchangeably with the vermilion from this point forward.

The lips are one of the most alluring features of a human face and contribute significantly to our perceptions of health, beauty, vitality, and how we interact with our environment. Much as other parts of the orofacial complex, they are exposed to pathology and weakening as a result of the aging process (loss of supportive collagen, elastin and extracellular matrix components); the constant contraction of the underlying orbicularis oris muscle; environmental insults (including smoking, UV radiation, extreme temperature) compounded by a lack of care.

Lips are the body's thinnest and most fragile layer of skin. Lip tissue is comprised of non‐keratinized epithelium, only 3 cell layers thick [3]. In addition, it has no sweat glands or sebaceous glands [4]. The lack of protective stratum corneum and natural moisturizing factors suggests that lips serve poorly as barriers. Their low moisture‐retaining capacity makes them prone to becoming dry and chapped, especially in drastic weather conditions [5, 6].

Although lip balms with a nourishing, moisturizing, and protective action are of help [7, 8], there has been an increasing need for novel therapies that hydrate the skin from the inside.

Polynucleotides (PN) are long‐chain polymers composed of nucleotide monomers, which are the basic building blocks of nucleic acids like DNA and RNA. Each nucleotide consists of a nitrogenous base, a sugar (ribose or deoxyribose), and a phosphate group. These units are linked via phosphodiester bonds to form a continuous backbone, resulting in single‐ or double‐stranded chains.

PN, typically derived from purified salmon DNA, has gained increasing popularity in the global aesthetic market with its exceptional biocompatibility [9, 10]. In addition to the distinct 3‐dimensional scaffold structure, which makes it suitable for skin rejuvenation [11], one of the key benefits of PN injection is increased skin hydration as it binds to water molecules and stimulates the body's natural hyaluronic acid (HA) production [12, 13].

The aim of this study was to determine if PN injection helps relieve lip dryness and assess safety in practicing PN on this specific area.

## 2 Materials and Methods

### 2.1 Study Design and Participants

**FIGURE 1:**
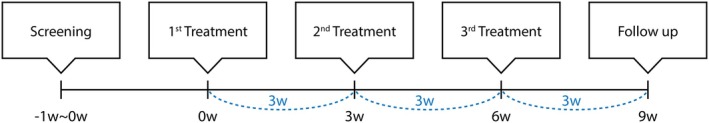
Study layout (Scheduled visits and assessment time points).

This was a multicenter, open‐label, prospective observational study. At visit 1 (week 0), individuals who desired improvement of their dry and chapped lips were screened, and eligible subjects gave written informed consent to participate in the study. The treatment consisted of a total of 3 injections (week 0, 3, and 6) where subjects received PN injection to the lips. A follow‐up visit was made 3 weeks after the 3rd injection (week 9) (Figure 1).

We enrolled healthy adults (aged ≥ 19 years) suffering from dry and chapped lips, as determined by a pretreatment Wrinkle Severity Rating Scale (WSRS) score of ≥ 2 points (1—“minimal”, 2—“slight”, 3—“moderate”, 4—“severe”, 5—“extreme lip wrinkles”) [14]. The exclusion criteria were as follows: (1) Pregnant or nursing women or women who plan to become pregnant during the study period; (2) Individuals suffering from a severe or progressive disease or any other pathology that may interfere with the study results; (3) History of allergy or anaphylactic shock, including hypersensitivity to PN and lidocaine; (4) Any type of injection to the lips within the past 6 months; and (5) Tendency to develop keloids or hypertrophic scars.

The study was conducted in accordance with good clinical practice, conforming to the ethical principles of the Declaration of Helsinki, and was approved by the Public Institutional Review Board (IRB number: P01‐202304‐01‐024).

### 2.2 Method of Administration and After Care

A highly purified PN product (REJURAN; PharmaResearch Inc., Seoul, Korea), which is a transparent sol–gel consisting of PN at a concentration of 20 mg/mL was used in this study.

**FIGURE 2:**
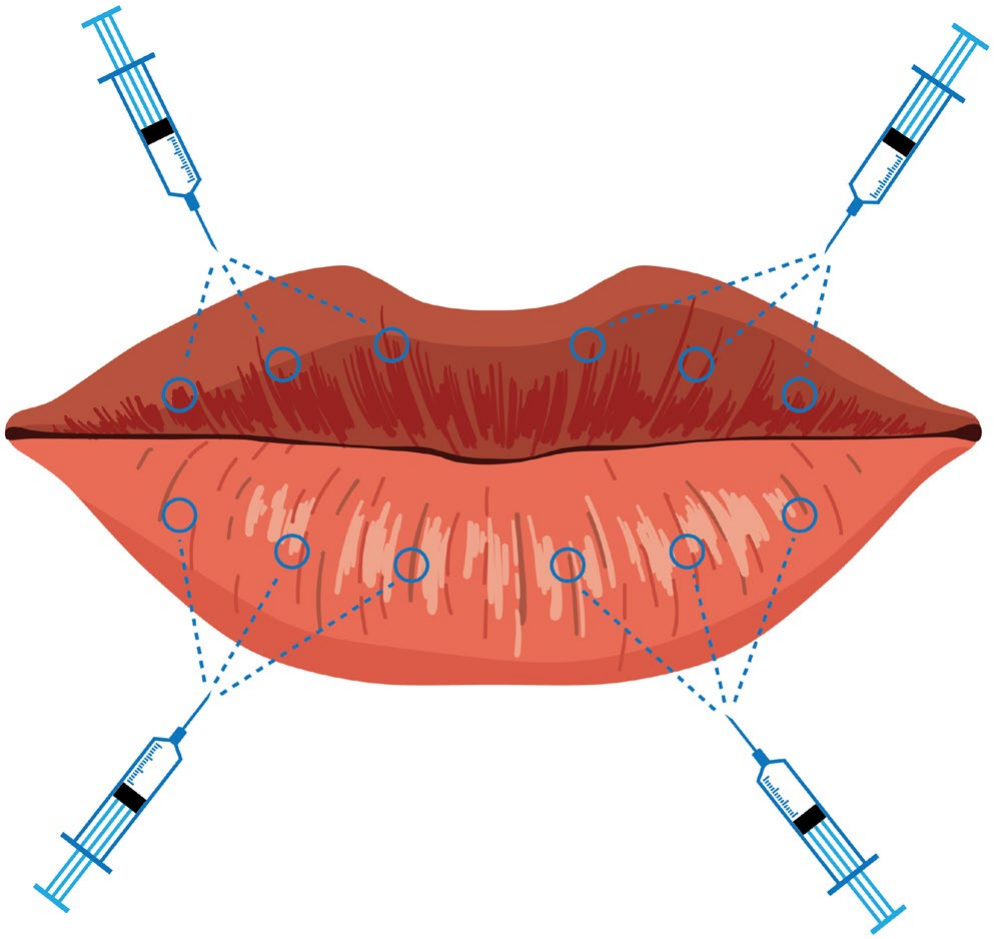
Points of administration. All treatments were performed using a multiple puncture technique, where equal amounts of PN were delivered subcutaneously at 12 points.

The procedure was performed by two investigators (N.K.R. and H.J.P.) with the patient under local anesthesia. A layer of anesthetic cream (EMLA, lidocaine 2.5% and prilocaine 2.5%, AstraZeneca AB, Sodertalje, Sweden) was applied over the treatment area for 30 min. All treatments were performed using a multiple puncture technique, where equal amounts of PN were delivered subcutaneously at 12 points (Figure 2). A volume of 1 mL was applied per session in all 3 sessions. A 4 mm, 33G hypodermic needle (JBP Nanoneedle, Feeltech, Jeollabuk‐do, Korea) was adopted.

**FIGURE 3:**
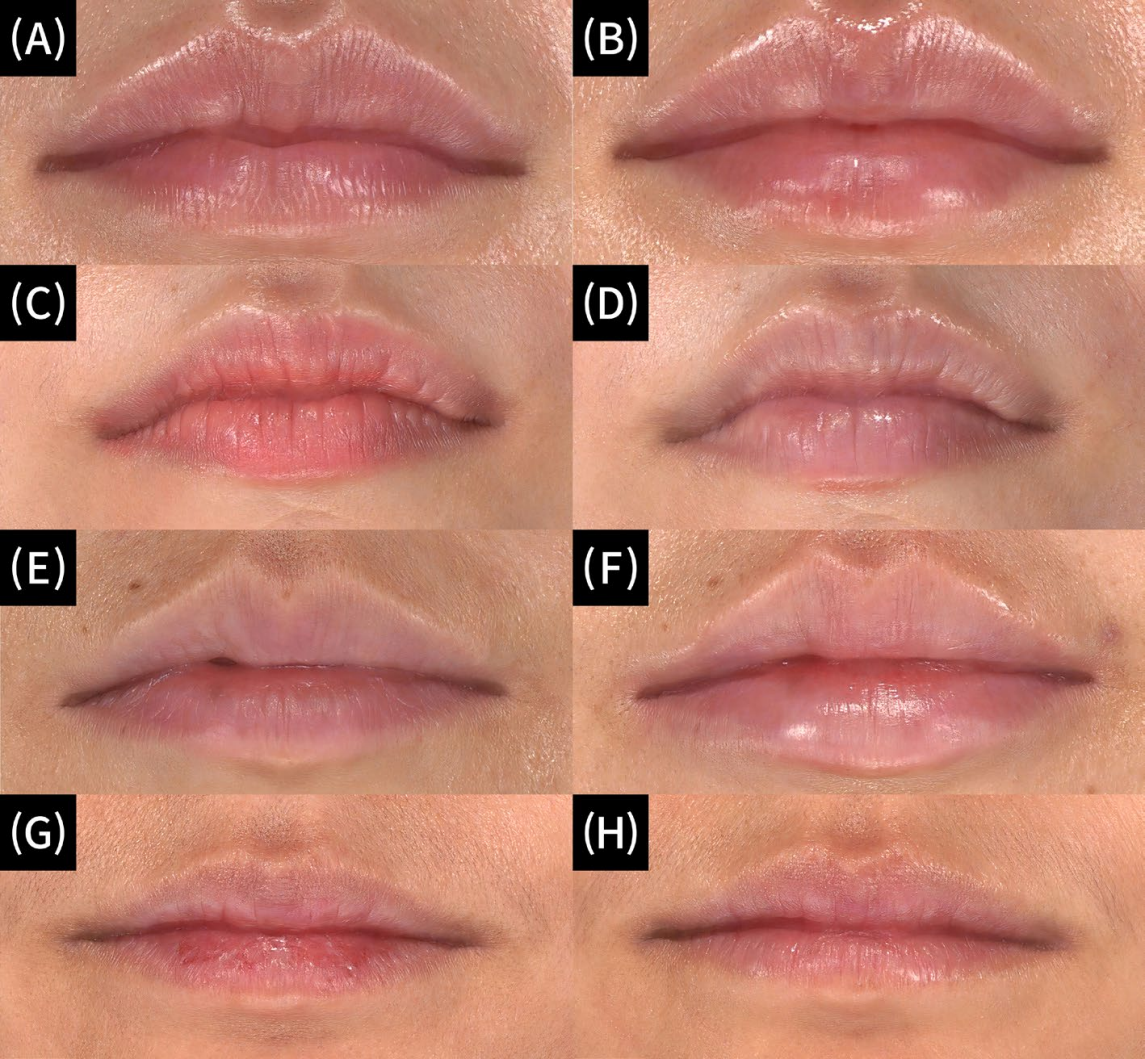
3D photos at baseline (A, C, E, G) and 3 weeks after the 3rd filler injection (B, D, F, H). The WSRS score changes are as follows: A 4 → B 1, C 4 → D 3, E 3 → F 1, G 3 → H 2. The LRGS score changes are as follows: A 3 → B 0, C 6 → D 2, E 4 → F 0, G 7 → H 1. WSRS score of ≥ 2 points (1—“minimal”, 2—“slight”, 3—“moderate”, 4—“severe”, 5—“extreme lip wrinkles”). LRGS scores (0—“No dryness or chapping evident”, 1‐2—“Slight, but definite roughness; fine scaling”, 3‐4—“Moderate roughness; coarse scaling; slight cracking, 5‐6—“Marked roughness; coarse scaling; obvious cracking, 7‐8—“Very marked roughness; coarse scaling; cracked progressing to fissuring).

To prevent or quickly alleviate transient side effects such as swelling and bruising, the treated area was cooled with an ice pack for 10–15 min. No analgesics were administered. Patients were instructed to avoid hot beverages, saunas, or baths on the day of treatment (Figure 3).

### 2.3 Clinical Assessment

Three‐dimensional (3D) images were taken before the procedures (including baseline), and at the follow‐up visit (3 weeks after the 3rd injection) with a facial skin analyzer (VECTRA M3, Canfield Scientific, Fairfield, NJ). Treatment efficacy was measured based on the WSRS and Lip Roughness Grading Scale (LRGS) scores (0—“No dryness or chapping evident”, 1‐2—“Slight, but definite roughness; fine scaling”, 3‐4—“Moderate roughness; coarse scaling”; slight cracking, 5‐6—“Marked roughness; coarse scaling; obvious cracking”, 7‐8—“Very marked roughness; coarse scaling; cracked progressing to fissuring”) [15] rated by the investigators as well as the 5—point Global Aesthetic Improvement Scale (GAIS) (3—“very much improved”, 2—“much improved”, 1—“improved”, 0—“no change”, −1—“worse”) scores from the study subjects at week 9. The safety and tolerability of PN were assessed from the adverse effect (AE) data collected at each visit. In particular, the Injection Site Reaction (ISR) assessment was conducted across a total of 9 items: swelling, firmness, tenderness, lumps, redness, pain, bruising, discoloration, and itching, 30 min after the first PN injection and at week 3 before PN injection.

### 2.4 Statistical Analysis

In our analysis, the primary endpoint was the WSRS score improvement rate at week 9 (Ratio of subjects whose WSRS score decreased by 1 point or more compared to baseline). The secondary endpoints were (1) Change in WSRS score at weeks 3, 6, and 9 compared to baseline, (2) Change in LRGS score at weeks 3, 6, and 9 compared to baseline, (3) LRGS score improvement rate at week 9 (Ratio of subjects whose LRGS score decreased by 1 point or more compared to baseline), (4) ISR assessment improvement rate at week 3 (loss/incidence × 100% for each item in the ISR assessment within 3 weeks), and (5) Ratio of subjects whose GAIS score was 1 point (“improved”) or above at week 9.

Continuous variables (i.e., age) are presented in mean (standard deviation, SD), whereas ordinal data are shown in median (interquartile range, IQR). The Wilcoxon's signed rank test was performed to examine the change in WSRS and LRGS scores at different time points. All statistical analyses conducted were performed using SPSS (Ver. 25, IBM Corporation, Armonk, NY, USA) with statistical significance at *p* < 0.05.

## 3 Results

A total of 30 individuals aged between 20 and 59 years (mean age of 35.1 ± 8.5 years; 29 (96.7%) female) were included in the study (Table 1). According to the WSRS and LRGS assessment conducted by the blinded independent evaluators, the participants initially had a median WSRS score of 3 (interquartile range, IQR 3–4) and a median LRGS score of 3 (IQR 2–6) for their lips.

**TABLE 1:** Clinical characteristics of the subjects at baseline.

Characteristics	*N* = 30
Age, years, mean (SD)	35.1 (8.5)
Age. years, *n* (%)	
< 30	8 (26.7)
30–39	14 (46.7)
40–49	6 (20)
≥ 50	2 (6.7)
Sex, *n* (%)	
Male	1 (3.3)
Female	29 (96.7)
WSRS, median (IQR)	
Initial (Pretreatment)	3 (3–4)
LRGS, median (IQR)	
Initial (Pretreatment)	3 (2–6)

Twenty‐seven participants completed the study. WSRS score improvement rate at week 9 (Ratio of subjects whose WSRS score decreased by 1 point or more compared to baseline) was 100%. A significant decrease in the median WSRS score (delta score, Δ) was observed following PN injection, beginning at week 3 (Δ −1), week 6 (Δ −1) and continuing up to week 9 (Δ −2) (*p* < 0.01, at all time points vs. baseline) (Table 2).

**TABLE 2:** Primary and secondary outcomes (WSRS score change, LRGS score change) after PN injection.

	Week 0 (Baseline)	Week 3 (Before 2nd injection)	Week 6 (Before 3rd injection)	Week 9 (At 3 week FU after Tx)
Injection with PN				
WSRS score Median (IQR)	3 (3–4)	2 (2–2)	2 (1–2)	1 (1–2)
WSRS score change Δ (IQR25%, IQR75%)	Reference	−1 (−1, −1)	−2 (−1, −2)	−2 (−1, −2)
*Z*	—	−4.613	−4.491	−4.647
*p*	—	< 0.01	< 0.01	< 0.01
LRGS score Median (IQR)	3 (2–6)	1 (1–3)	1 (1–2)	1 (0–2)
LRGS score change Δ (IQR 25%, IQR75%)	Reference	−1 (−1, −3)	−2 (−1, −4)	−3 (−1, −4)
*Z*	—	−4.430	−4.429	−4.313
*p*	—	< 0.01	< 0.01	< 0.01

A significant decrease in the median LRGS score (delta score, Δ) was observed following PN injection, beginning at week 3 (Δ −1), week 6 (Δ −2) and continuing up to week 9 (Δ −3) (*p* < 0.01, at all time points vs. baseline) (Table 2). LRGS score improvement rate at week 9 (Ratio of subjects whose LRGS score decreased by 1 point or more compared to baseline) was 89%.

ISR assessment 30 min after the first PN injection are as follows: number of participants who developed swelling (*n* = 27, 100%), firmness (*n* = 7, 26%), tenderness (*n* = 2, 7%), lumps (*n* = 6, 22%), redness (*n* = 21, 78%), pain (*n* = 24, 89%), bruising (*n* = 6, 22%), discoloration (*n* = 0, 0%), and itching (*n* = 1, 4%) (Table 3). Most were mild and transient, resolving on the same day within a few hours. ISR assessment improvement rate at week 3 (loss/incidence x 100% for each item in the ISR assessment within 3 weeks) are as follows: swelling (93%), firmness (100%), tenderness (100%), lumps (100%), redness (95%), pain (100%), bruising (100%), itching (100%) (Table 3).

**TABLE 3:** ISR assessment 30 min after the 1st PN injection and at week 3 (before the 2nd PN injection).

IRS	Baseline		Week 3		*p*
	Incidence		Resolved		
	*N*	%	*N*	%	
Swelling	27	100	25	93	< 0.05
Firmness	7	26	7	100	< 0.05
Tenderness	2	7	2	100	*
Lumps	6	22	6	100	< 0.05
Redness	21	78	20	95	< 0.05
Pain	24	89	24	100	< 0.05
Bruising	6	22	6	100	< 0.05
Discoloration	0	0	—	—	—
Itching	1	4	1	100	*

*Note:* * means that the number of ISR incidence is too small to recognize statistical significance in ISR resolution at week 3.

The median GAIS scores from the study subjects at week 9 were 2 (IQR 1–3). The Ratio of subjects whose GAIS score was 1 point (“improved”) or above at week 9 was 100%.

## 4 Discussion

The term “skin booster” was first used interchangeably with small particle HA but now includes a variety of injectable substances that enhance the skin's overall condition [9, 16]. Among the various skin boosters, PN stands out from other biostimulators as it is sourced from a natural origin rather than being a synthetic polymer‐based product [17]. Currently, a number of PN injectables are available in different countries (i.e., NEWEST and PLINEST, Mastelli, Italy) with REJURAN (PharmaResearch Inc., Seoul, Korea) being the first commercially available highly purified PN product [18].

The main advantage of the highly purified PN is the high water‐binding capacity [13] as well as its trophic action on dermal fibroblasts [19, 20]. Such features allow PN to plump the extracellular matrix with a boost in skin elasticity and turgor [10, 11, 12, 17, 21]. Accordingly, a survey on Korean dermatologists identified the top 6 indications for injectable PN to be fine lines on the cheeks followed by infraorbital fine lines, periorbital fine lines, uneven skin texture, dry skin, and fine lines on the forehead [18].

Lips are often overlooked when it comes to skincare, but they deserve just as much attention as the rest of our face. While prior studies of the lip mostly focused on age‐related changes rather than functional problems, dryness is the most common lip complaint [6, 7]. Since dry and chapped lips display fine lines (wrinkles) and textural changes (roughness) from the flaking and peeling of corneocytes, we adopted the WSRS and the LRGS to assess lip dryness [14, 15]. Skin dryness is often determined by measuring the trans‐epidermal water loss (TEWL) (i.e., the higher the TEWL value, the drier the skin). However, TEWL may not truly reflect the moisturizing function of the lip as exemplified by the low TEWL in dry lips with abnormal desquamation [6, 22, 23].

Based on the aforementioned properties of PN, we assumed that REJURAN injection would have abeneficial role in relieving dry and chapped lips, which was confirmed by our study findings.

All 30 subjects enrolled in this study were Korean with only 1 man, which denotes women's greater interest in their appearance. Approximately half of the participants were in their 30s, followed by individuals in their 20s, which indicates that dry lips are not limited to the aged population. A Japanese study has in fact reported that women in their 30s have dry and chapped lips most frequently, whereas problems associated with the shape and color of the lip increase with age [24].

Both the WSRS and LRGS scores continuously improved over repeated PN injections, supporting the need for multiple injections for dry and chapped lips. Not surprisingly, a minimum of 3 sessions of PN injection, each spaced 3–4 weeks apart, is a routine cosmetic practice among Korean dermatologists [18]. While the WSRS score improvement rate at week 9 was 100%, the LRGS score improvement rate at week 9 was 89%, indicating that it likely takes more time and additional PN injections to attenuate lip roughness and scales than fine lines.

The most common ISR from our participants was swelling (100%), followed by pain (89%), and redness (78%). With the abundance of polar functional groups, PN can attract and permanently bind water molecules [13, 25]. According to a meta‐analysis, the 5 most common side effects from lip augmentation with HA were injection site tenderness (88.7%), swelling (74.3%), contusion (48.7%), mass (27.3%), and pain (19.7%) [26]. This documentation of AEs is not optimal for direct comparison with variation in the injected substance (PN vs HA) and injection volume tailored to different indications (dry and chapped lips vs. lip augmentation). Unfortunately, no studies are available on the effectiveness of lip hydration (and concurrent AEs) in response to HA injection. One of the most noticeable differences in ISR from PN to HA was the higher incidence of pain, which can be troublesome. PN injection is well known to accompany a fair amount of discomfort, largely due to the introduction of a high molecular weight viscoelastic substance into the superficial layer of the skin [18]. Much effort has been made to overcome this problem, such as the introduction of a PN product containing lidocaine. As for this observational study, the original product (REJURAN) was injected, but in actual practice, we recommend using REJURAN HB, which contains 0.3% lidocaine, to maximize patient comfort.

There are some limitations to this study. First, the participants were exclusively Korean, which restricts the generalizability of our findings. In addition to the degree of lip dryness [27], there is an ethnic difference in the perception of an ideal lip as well as lip aging where Asians prefer a smaller lip and are less likely to develop perioral fine lines while retaining their lip volume [28, 29, 30, 31]. Koreans are also becoming wary of cosmetic “filler” products with growing awareness of serious filler complications. Such characteristics and trends may make PN a favorable choice for lip dryness and chapping over HA fillers in Korea. Second, we did not measure the capacitance of the lip. Third, we were unable to assess and survey the 3 individuals who dropped out from the study. Lastly, a control group using standard treatments such as a lip balm was not included due to the preliminary nature of the study, lack of a standardized comparator, distinct mechanism of action of PN, and ethical and practical considerations.

Despite these potential biases, our results indicate that PN may be a viable option for dry and chapped lips. Since this is the very first study to explore the potential of PN on lip hydration, further studies involving a larger number and a wider range of ethnicities, as well as longer follow‐up, are necessary to confirm our findings. We also plan to include comparative arms with standard topical agents to further delineate the relative efficacy and patient‐reported outcomes.

## Conflicts of Interest

The authors declare no conflicts of interest.

## Data Availability

The data that support the findings of this study are available on request from the corresponding author. The data are not publicly available due to privacy or ethical restrictions.

## References

[1] J. Shang , X. Feng , Y. Chen , Z. Gu , and Y. Liu , “Human Lip Vermilion: Physiology and Age‐Related Changes,” Journal of Cosmetic Dermatology 23, no. 8 (2024): 2676–2680, 10.1111/jocd.16317. 38590116

[2] C. Zugerman , “The Lips: Anatomy and Differential Diagnosis,” Cutis 38, no. 2 (1986): 116–120. 3743125

[3] Z. Ya‐Xian , T. Suetake , and H. Tagami , “Number of Cell Layers of the Stratum Corneum in Normal Skin ‐ Relationship to the Anatomical Location on the Body, Age, Sex and Physical Parameters,” Archives of Dermatological Research 291, no. 10 (1999): 555–559, 10.1007/s004030050453. 10552214

[4] H. Kobayashi and H. Tagami , “Functional Properties of the Surface of the Vermilion Border of the Lips Are Distinct From Those of the Facial Skin,” British Journal of Dermatology 150, no. 3 (2004): 563–567, 10.1046/j.1365-2133.2003.05741.x. 15030342

[5] N. S. Trookman , R. L. Rizer , R. Ford , R. Mehta , and V. Gotz , “Clinical Assessment of a Combination Lip Treatment to Restore Moisturization and Fullness,” Journal of Clinical and Aesthetic Dermatology 2, no. 12 (2009): 44–48. PMC2923945 20725584

[6] J. Kim , H. Yeo , T. Kim , E. T. Jeong , J. M. Lim , and S. G. Park , “Relationship Between Lip Skin Biophysical and Biochemical Characteristics With Corneocyte Unevenness Ratio as a New Parameter to Assess the Severity of Lip Scaling,” International Journal of Cosmetic Science 43, no. 3 (2021): 275–282, 10.1111/ics.12692. 33544395 PMC8251770

[7] E. Tamura , J. Ishikawa , Y. Yasuda , and T. Yamamoto , “The Efficacy of Synthetic Pseudo‐Ceramide for Dry and Rough Lips,” International Journal of Cosmetic Science 43, no. 2 (2021): 158–164, 10.1111/ics.12677. 33258166 PMC8252384

[8] E. Tamura , H. Yasumori , and T. Yamamoto , “The Efficacy of a Highly Occlusive Formulation for Dry Lips,” International Journal of Cosmetic Science 42, no. 1 (2020): 46–52, 10.1111/ics.12583. 31571236

[9] K. H. Yi , W. Winayanuwattikun , S. Y. Kim , et al., “Skin Boosters: Definitions and Varied Classifications,” Skin Research and Technology 30, no. 3 (2024): e13627, 10.1111/srt.13627. 38481069 PMC10938033

[10] K. Y. Park , J. Seok , N. K. Rho , B. J. Kim , and M. N. Kim , “Long‐Chain Polynucleotide Filler for Skin Rejuvenation: Efficacy and Complications in Five Patients,” Dermatologic Therapy 29, no. 1 (2016): 37–40, 10.1111/dth.12299. 26814448

[11] Y. J. Lee , H. T. Kim , Y. J. Lee , et al., “Comparison of the Effects of Polynucleotide and Hyaluronic Acid Fillers on Periocular Rejuvenation: A Randomized, Double‐Blind, Split‐Face Trial,” Journal of Dermatological Treatment 33, no. 1 (2022): 254–260, 10.1080/09546634.2020.1748857. 32248707

[12] M. Cavallini , E. Bartoletti , L. Maioli , et al., “Consensus Report on the Use of PN‐HPT (Polynucleotides Highly Purified Technology) in Aesthetic Medicine,” Journal of Cosmetic Dermatology 20, no. 3 (2021): 922–928, 10.1111/jocd.13679. 32799391 PMC7984045

[13] M. Cavallini and M. Papagni , “Long Chain Polynucleotides Gel and Skin Biorevitalization,” Journal of Plastic Dermatology 3 (2007): 27–32.

[14] J. S. Ryu , S. G. Park , T. J. Kwak , et al., “Improving Lip Wrinkles: Lipstick‐Related Image Analysis,” Skin Research and Technology 11, no. 3 (2005): 157–164, 10.1111/j.1600-0846.2005.00115.x. 15998326

[15] C. F. Gfeller , R. Wanser , H. Mahalingam , et al., “A Series of In Vitro and Human Studies of a Novel Lip Cream Formulation for Protecting Against Environmental Triggers of Recurrent Herpes Labialis,” Clinical, Cosmetic and Investigational Dermatology 12 (2019): 193–208, 10.2147/ccid.S179430. 30962701 PMC6432897

[16] G. Arora , S. Arora , R. Sadoughifar , and N. Batra , “Biorevitalization of the Skin With Skin Boosters: Concepts, Variables, and Limitations,” Journal of Cosmetic Dermatology 20, no. 8 (2021): 2458–2462, 10.1111/jocd.13871. 33249741

[17] K. W. A. Lee , K. W. L. Chan , A. Lee , et al., “Polynucleotides in Aesthetic Medicine: A Review of Current Practices and Perceived Effectiveness,” International Journal of Molecular Sciences 25, no. 15 (2024): 8224, 10.3390/ijms25158224. 39125793 PMC11311621

[18] N. K. Rho , K. H. Han , M. Cho , and H. S. Kim , “A Survey on the Cosmetic Use of Injectable Polynucleotide: The Pattern of Practice Among Korean Dermatologists,” Journal of Cosmetic Dermatology 23, no. 4 (2024): 1243–1252, 10.1111/jocd.16125. 38093498

[19] P. Sini , A. Denti , G. Cattarini , M. Daglio , M. E. Tira , and C. Balduini , “Effect of Polydeoxyribonucleotides on Human Fibroblasts in Primary Culture,” Cell Biochemistry and Function 17, no. 2 (1999): 107–114. 10377956 10.1002/(SICI)1099-0844(199906)17:2<107::AID-CBF815>3.0.CO;2-#

[20] S. Thellung , T. Florio , A. Maragliano , G. Cattarini , and G. Schettini , “Polydeoxyribonucleotides Enhance the Proliferation of Human Skin Fibroblasts: Involvement of A2 Purinergic Receptor Subtypes,” Life Sciences 64, no. 18 (1999): 1661–1674, 10.1016/s0024-3205(99)00104-6. 10328526

[21] G. J. Jeong , G. R. Ahn , S. J. Park , J. Y. Hong , and B. J. Kim , “A Randomized, Patient/Evaluator‐Blinded, Split‐Face Study to Compare the Efficacy and Safety of Polycaprolactone and Polynucleotide Fillers in the Correction of Crow's Feet: The Latest Biostimulatory Dermal Filler for Crow's Feet,” Journal of Cosmetic Dermatology 19, no. 7 (2020): 1593–1599, 10.1111/jocd.13199. 31680395

[22] E. Tamura , J. Ishikawa , A. Naoe , and T. Yamamoto , “The Roughness of Lip Skin Is Related to the Ceramide Profile in the Stratum Corneum,” International Journal of Cosmetic Science 38, no. 6 (2016): 615–621, 10.1111/ics.12335. 27090066

[23] R. Hikima , S. Igarashi , N. Ikeda , et al., “Development of Lip Treatment on the Basis of Desquamation Mechanism,” International Journal of Cosmetic Science 26, no. 3 (2004): 165, 10.1111/j.1467-2494.2004.00217_01.x.

[24] E. Tamura , J. Ishikawa , K. Sugata , K. Tsukahara , H. Yasumori , and T. Yamamoto , “Age‐Related Differences in the Functional Properties of Lips Compared With Skin,” Skin Research and Technology 24, no. 3 (2018): 472–478, 10.1111/srt.12456. 29405429

[25] M. T. Colangelo , P. Govoni , S. Belletti , F. Squadrito , S. Guizzardi , and C. Galli , “Polynucleotide Biogel Enhances Tissue Repair, Matrix Deposition and Organization,” Journal of Biological Regulators and Homeostatic Agents 35, no. 1 (2021): 355–362, 10.23812/20-320-l. 33480222

[26] L. M. Czumbel , S. Farkasdi , N. Gede , et al., “Hyaluronic Acid Is an Effective Dermal Filler for Lip Augmentation: A Meta‐Analysis,” Frontiers in Surgery 8 (2021): 681028, 10.3389/fsurg.2021.681028. 34422892 PMC8377277

[27] H. B. Gunt and S. B. Levy , “Effect of Race on Lower Lip Hydration,” Journal of Clinical and Aesthetic Dermatology 17, no. 1 (2024): 28–32. PMC10826831 38298747

[28] C. Subramanyam , H. B. Gunt , and R. K. Sivamani , “Clinical Features and Biophysical Characteristics of Lips of South Asian Women,” Clinical, Cosmetic and Investigational Dermatology 16 (2023): 1955–1961, 10.2147/ccid.S417214. 37525690 PMC10387256

[29] A. Ding , “The Ideal Lips: Lessons Learnt From the Literature,” Aesthetic Plastic Surgery 45, no. 4 (2021): 1520–1530, 10.1007/s00266-021-02190-x. 33649926

[30] P. I. Heidekrueger , C. Szpalski , K. Weichman , et al., “Lip Attractiveness: A Cross‐Cultural Analysis,” Aesthetic Surgery Journal 37, no. 7 (2017): 828–836, 10.1093/asj/sjw168. 27677824

[31] G. Lemperle , R. Anderson , and T. R. Knapp , “An Index for Quantitative Assessment of Lip Augmentation,” Aesthetic Surgery Journal 30, no. 3 (2010): 301–310, 10.1177/1090820x10374095. 20601553

